# Dye stability of black cherry plum anthocyanins in the interaction with co‐pigments and sucrose sweetener

**DOI:** 10.1002/fsn3.3943

**Published:** 2024-01-07

**Authors:** Shahrbanu Molaeafard, Rashid Jamei, Ahmad Poursattar Marjani

**Affiliations:** ^1^ Department of Biology, Faculty of Basic Sciences and Chemistry Urmia University Urmia Iran; ^2^ Department of Organic Chemistry, Faculty of Chemistry Urmia University Urmia Iran

**Keywords:** anthocyanin, Co‐pigmentation, furfural, HPLC, *Prunus cerasifera* Ehrh. cv. Pissardii Nigra, thermal stability

## Abstract

In this research, the co‐pigmentation reactions between black cherry plum (*Prunus cerasifera* Ehrh. cv. Pissardii Nigra) anthocyanins and caffeic, gallic, 4‐hydroxybenzoic, malic, and tannic acids with different concentrations (0, 120, 240, 480, and 960 mg L^−1^) at various temperatures (20, 40, 60, 80, and 100°C) were investigated at pH 3.5. The strongest immediate co‐pigmentations resulted at 960 mg L^−1^, being significantly highest using tannic acid at all temperatures. In addition, the anthocyanin stability and the brown polymeric color formation were investigated in the presence of different concentrations of sucrose sweetener (0, 30, and 60%) and different pHs (2 and 3) in the range of 0–60 h. Also, the amount of furfural was measured in the presence of 0% and 30% sucrose concentrations at pH 2 after 20 h at 90°C by HPLC (high‐performance liquid chromatography), and the most polymeric color formation was observed in the concentration of 60% sucrose at pH 2 after 60 h.

## Graphical abstract

In this study, the effect of different co‐pigments on anthocyanin stability at 20, 40, 60, 80, and 100°C and sucrose sweetener on anthocyanin degradation at 90°C in black cherry plum fruits was investigated. Tannic acid caused the strongest effect on anthocyanin stability, especially at the concentration of 960 mg L^−1^, and the highest polymeric color formation was observed in the concentration of 60% sucrose at pH 2 after 60 h.

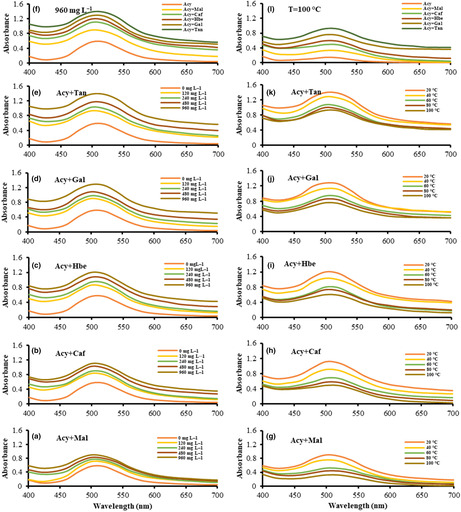

## 1 INTRODUCTION

The appearance of food on store shelves is often very important for the customer's choice. Synthetic dyes have been used as an alternative to natural ones because of some properties, such as high stability. However, the tendency to replace them with natural dyes has increased, because of their positive effects on health, such as anti‐bacterial and anti‐inflammatory activity. Anthocyanins are mainly responsible for the red, purple, pink, and blue colors of vegetables, fruits, grains, and flowers (Rodriguez‐Amaya, 2016). The high sensitivity of anthocyanins to environmental factors like oxygen, pH, light, temperature, ascorbic acid, and enzymes causes some issues like bioactivity loss and color fading during different processing and storage stages in the food industry (Cavalcanti et al., 2011). Generally, the co‐pigmentation reactions can cause enhanced and stabilized anthocyanin pigments. Organic acids, metals, flavonoids, or other anthocyanins can be used as co‐pigments (Castañeda‐Ovando et al., 2009). Non‐covalent interactions such as hydrogen bonding are very important in this process; hence, any factor affecting their formation, like proton donors and acceptors or aromatic rings, is significant (Lambert et al., 2011). Quantitative processes in addition to qualitative processes have been investigated during spectrophotometric studies of intermolecular co‐pigmentation reactions between anthocyanins and various classes of natural sources, as well as the role of some factors such as temperature, pH, concentration, and solvent type on the magnitude of co‐pigmentation (Türkyılmaz et al., 2019; Zhang et al., 2015).

Besides, heat treatment is associated with the loss of anthocyanins during food processing. The high content of saccharides, including sucrose, and high temperature lead to furfural formation and browning processes through the two possible pathways of caramelization and Maillard, which lead to the loss of anthocyanin pigments. The hydrolysis of sucrose is the first reaction step in the caramelization reaction, which happens with the protonation of the glycosidic bond. The proton used in this step of the reaction can be derived from water (or even sucrose) dissociation at high temperatures, as well as from acidic reaction products (Jimenez et al., 2010; Quintas et al., 2007; Tsai et al., 2005). Ertan et al. (2018) reported that the stability of anthocyanins and color density in sour cherry nectar sweetened with honey were higher than those sweetened with sucrose and maltose syrup.

There are different hybrids mainly with purple foliage: e.g., ‘Pissardii’ with white flowers and purple leaves (*P. cerasifera* Ehrh. cv. Pissardii), and ‘Nigra’ smaller plants whit pink flowers and deep red‐purple leaves (*P. cerasifera* Ehrh. cv. Pissardii Nigra) (Black/Purple cherry plum) (Szekely & Dagmar, 2011). The fruits of the red and purple varieties of cherry plum are rich in anthocyanins, including cyanidin‐3‐galactoside, cyanidin‐3‐glucoside, cyanidin‐3‐rutinoside, and cyanidin (acetyl)‐3‐glucoside. These fruits, especially their peels, could be used as a resource for natural pigment extraction (Wang et al., 2012).

The present study focuses on the effect of different colorless co‐pigments (tannic acid, gallic acid, 4‐hydroxybenzoic acid, caffeic acid, and malic acid) on anthocyanin stability, as well as the effect of sucrose sweetener on anthocyanin degradation and brown polymer color formation. The magnitudes of the hyperchromic effects, bathochromic shifts, and brown polymeric color formation were all investigated spectrophotometrically, and the amount of furfural was measured by HPLC (high‐performance liquid chromatography).

## 2 MATERIALS AND METHODS

### 2.1 Chemicals

Sucrose, caffeic, gallic, 4‐hydroxy benzoic, tannic, and malic acids were procured from Merck (Darmstadt, Germany), and phosphoric acid (85%), sodium acetate (CH_3_COONa), potassium chloride, HCl (37%), and sodium hydroxide were procured from Sigma–Aldrich companies (St. Louis, USA).

### 2.2 Anthocyanin extraction and purification

One kilogram of the black cherry plum (BCP) fruits (fleshes with skins) was procured from Urmia Gardens with herbarium number 6160, determined by the Biology Department of Urmia University. The fruits were washed with distilled water and disinfected with fruit disinfectant, and the anthocyanins were extracted from the flesh and skin of fruits with ethanol 96% (acidified with 0.1% HCl) under stirring for 3 h, followed by centrifugation at 8000*g* for half hour by Centurion Scientific's K280R Centrifuge (UK) (Wrolstad et al., 2005). The residue was re‐extracted by ethanol solvent until its discoloration, and the supernatant was filtered with Whatman No. 1 filters by a Büchner funnel 3 times. Purification of anthocyanins was performed by ion‐exchange chromatography using a cationic Dowex resin column (Dowex Monosphere 650 C) (Narayan & Venkataraman, 2000). The purified anthocyanin extract was concentrated by the rotary evaporator (Büchi, Germany) under reduced pressure at 30–35°C. The remaining solid extract (219.4 g) from the rotary evaporator was completely dissolved in double‐distilled water, and its volume was increased to 1000 mL by double‐distilled water. The monomeric anthocyanin content of BCP extract was 31.34 ± 0.251 mg kg^−1^, determined by the pH‐differential methods (Lee et al., 2005). Thirty gram of BCP fruits were used to extract anthocyanin for HPLC analysis using methanol: water: formic acid solvent with a ratio of 70:28:2, and the filtered extract (prepared using the method mentioned above) was used for furfural measurement (Chen et al., 2013).

### 2.3 Co‐pigmentation treatment

The experiments on the intermolecular co‐pigmentation process were carried out following the methods described by Malaj et al. (2013), Sun et al. (2010), and Babaloo and Jamei (2018), with minor changes. Co‐pigment buffer was prepared using H_2_SO_4_ (3.5 mL, 0.06 M) and CH_3_COONa (1.64 g, 0.02 M), and its pH was adjusted to 3.5 by adding HCl. Five co‐pigmented samples, i.e., Acy + Caf, Acy + Gal, Acy + Hbe, Acy + Tan, and Acy + Mal, were prepared at a molar ratio of 1:1 at five concentrations of them (0, 120, 240, 480, and 960 mg L^−1^) at 20°C. The effect of increasing temperature conditions was studied in the range of 20–100°C (intervals of 20°) using a water bath (Memmert, German) at the highest concentration of co‐pigments (960 mg L^−1^). All absorption spectra were scanned by a spectrophotometer. Hyperchromic effects (Δ*A*_vis‐max_) were measured as an increase in the absorbance values at *A*_vis‐max_, and bathochromic shifts (Δ*λ*_vis‐max_) were measured as a rise in shift values at *λ*_vis‐max_ (nm).

### 2.4 Sucrose treatment

Two different concentrations of sucrose (30% and 60%) at two pHs (2 and 3) at 90°C during a period of 0–60 h were investigated on BCP anthocyanin samples, and 30 mL of extracted anthocyanins were adjusted to pH 2 and pH 3 by citrate buffer. The samples were transferred to capped test tubes and placed inside a water bath previously set at 90°C (Tsai et al., 2005).

### 2.5 Furfural measurement by HPLC

The rate of furfural of Suc 30%, Acy + 0% Suc, and Acy + 30% Suc samples was measured by HPLC (KNAUER, Germany) after 20 h of heating at 90°C at pH 2. One milliliter of BCP anthocyanin samples was mixed with 1 mL of 1, 2‐dichloromethane for 5 min, and this caused the formation of two lower (colorless) and upper (yellow) phases. The upper phase, containing furfural, was used for HPLC. A furfural standard was used for furfural measurement (Liu et al., 2014). Column length, inner diameter, and particle size of the Eurospher C18 column were 25 cm, 4.6 μm, and 5 μm, respectively. Acetonitrile:water with ratios of 92:8 was used for the mobile phase, and the flow rate was considered to be 1 mL/min. The detector was set to 280 nm due to the absorption of furfural at this wavelength (Tsai et al., 2005).

### 2.6 Statistical analysis

Mean values were obtained from three replications for each experiment. The data was analyzed using SPSS 26, and the one‐way ANOVA Tukey test with a probability of *p* ≤ .05 was utilized to determine statistical differences among means. Then, diagrams were plotted with Microsoft Excel 2016.

## 3 RESULTS AND DISCUSSION

### 3.1 Co‐pigmentation as a function of increasing co‐pigment concentration

**FIGURE 1:**
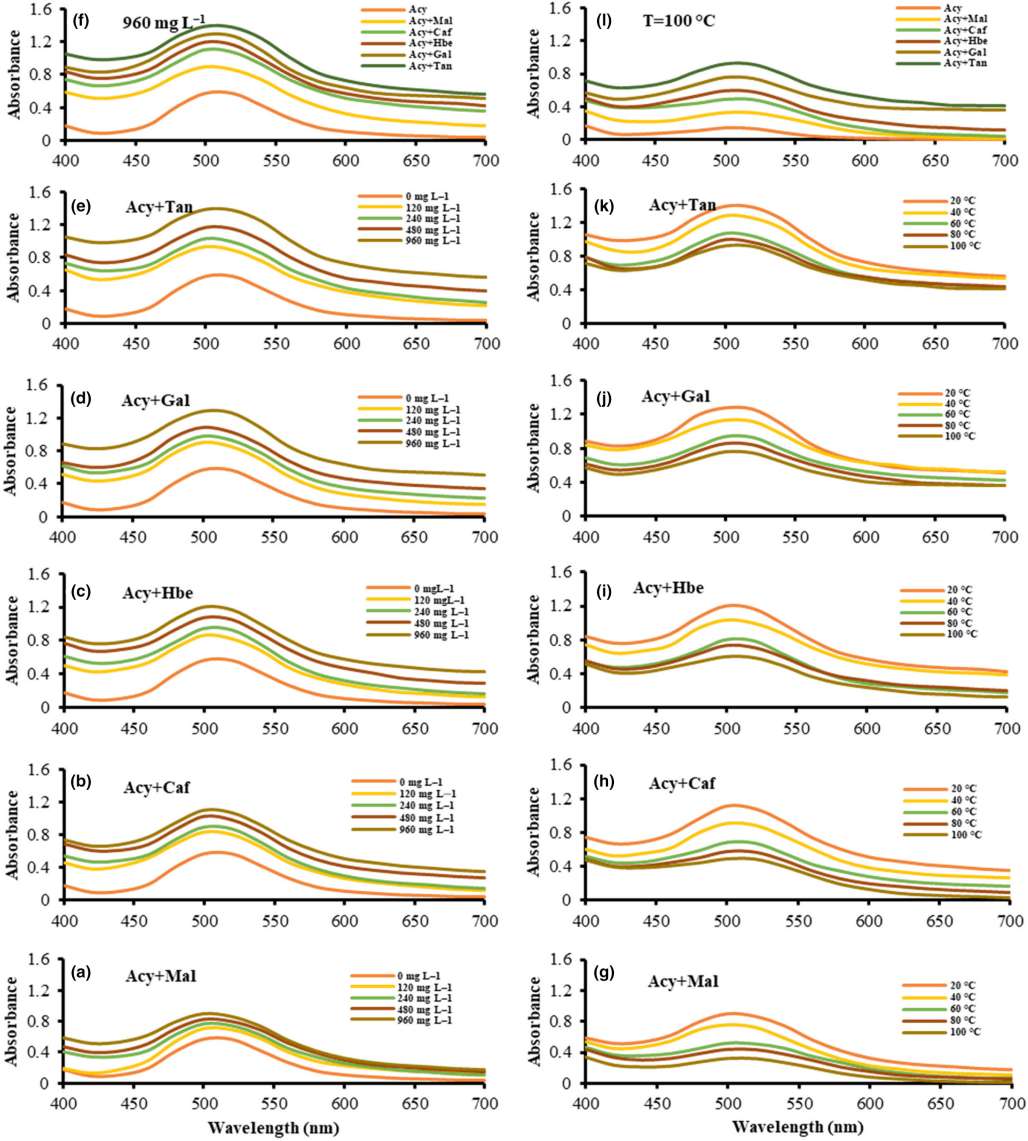
Visible absorption spectra of natural and co‐pigmented anthocyanins with various concentrations of co‐pigments in the range of 120–960 mg L^−1^ at 20°C (a–e); Absorption spectra of natural and various co‐pigmented anthocyanins at the concentration of 960 mg L^−1^ (f); Visible absorption spectra of natural and co‐pigmented anthocyanins with different temperature conditions in the range of 20–100°C at the concentration of 960 mg L^−1^ (g–k); Absorption spectra of natural and various co‐pigmented anthocyanins at 100°C (l).

The co‐pigmentation effect of five co‐pigments on the anthocyanins of BCP extract was investigated at pH 3.5. The concentration of anthocyanin was constant during the experiments, and five increasing concentrations (0, 120, 240, 480, and 960 mg L^−1^) of various co‐pigments in the molar ratio of 1:1 with BCP anthocyanins were prepared for intermolecular co‐pigmentation reactions. Figure 1a–e shows the magnitude of the hyperchromic effects in the presence of co‐pigments with increasing concentrations by indicating the increase in absorbance spectra in comparing natural anthocyanin at the range of 400–700 nm. The concentrations of 960 and 120 mg L^−1^ caused the highest and lowest hyperchromic effect, respectively, in all anthocyanin‐co‐pigment complexes, and the absorption spectrum of natural anthocyanins was lower than of all co‐pigmented anthocyanins. Therefore, the co‐pigmentation in all concentrations enhanced the color intensity of anthocyanins. Different effects were also observed in the presence of various co‐pigments. Figure 1f indicates the plots of visible spectra of various co‐pigmented samples in comparison with the natural anthocyanin on hyperchromic effect at 960 mg L^−1^. The visible spectra indicated that the best color intensity was observed in Acy + Tan complex, followed by the Acy + Gal, Acy + Hbe, Acy + Caf, and Acy + Mal complexes, respectively. Babaloo and Jamei (2018) suggested the concentration of 960 mg L^−1^ as the best effective concentration on color intensity among the concentrations of 120, 240, and 480 mg L^−1^ of tannic, caffeic, coumaric, and benzoic acids at a 1:1 molar ratio with anthocyanin extracted from blueberry (*Cornus mas‐*Macrocarpa) fruits at pH 3.5.

**FIGURE 2:**
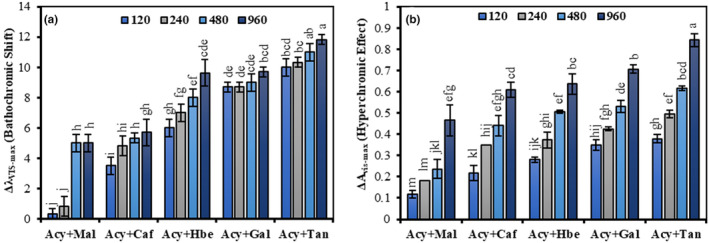
The magnitude of bathochromic shift (a) and hyperchromic effect (b) during co‐pigmentation in the presence of various co‐pigments with different concentrations (120–960 mg L^−1^).

Bathochromic shifts in the *A*_vis‐max_ were also observed in all the studied complexes (five Acy + co‐pigment complexes) because of the intermolecular co‐pigmentation reactions (Figure 2a). Figure 2a,b shows the increase of absorbance (Δ*A*_vis‐max_) and wavelength (Δ*λ*_vis‐max_) with increasing co‐pigment amount, and the magnitudes of the hyperchromic effects and the bathochromic shifts were observed on 0.118–0.843 units and 0.33–11.83 nm, respectively, on different co‐pigment amounts (120–960 mg L^−1^). It was affirmed that the highest concentration of these co‐pigments (960 mg L^−1^) strongly influences both hyperchromic and bathochromic shifts in the order of Tan > Gal > Hbe > Caf > Mal. In natural anthocyanins, no hyperchromic effects and bathochromic shifts were observed after the half hour.

The Δ*A*_vis‐max_ and Δ*λ*_vis‐max_ can be observed due to the hydration equilibrium shift toward the cationic form, which is illustrated by the following equations according to Kanha et al. (2019), Brouillard et al. (1989), and Zhang et al. (2015):

(1) $${\mathrm{AH}}^{+}\overset{K_{a}}{⇌}\;A+H^{+},$$

(2) $${\mathrm{AH}}^{+}+H_{2}O\;\overset{K_{h}}{⇌}\;\left(B+C_{E}\right)+H^{+},$$

(3) $$\left(B+C_{E}\right)\;\overset{K_{T}}{⇌}\;C_{Z},$$

(4) $${\mathrm{AH}}^{+}+{\mathrm{nC}}_{P}\;\overset{K}{⇌}\;\mathrm{AH}{\left(C_{P}\right)}_{n}^{+}.$$

The equilibrium reactions of natural anthocyanin in the aqueous solution conditions can be summarized by equations (1)–(3) at the pH range of about 3–4. At this pH range, B (hemiketal) and the C_E_ (*cis*‐chalcone) are dominant forms, in rapid equilibrium together. Also, in this pH range, the AH^+^ (red flavylium cation) is found in small quantities, while C_Z_ (*trans*‐chalcone) and A (blue quinoidal base) forms can also be present in lower quantities. The addition of each co‐pigment in the anthocyanin extract causes the equilibrium shift of equation (2) and the occurrence of equation (4), which leads to the formation of flavylium ions because of the interaction between colorless co‐pigments and flavylium cation forms of BCP anthocyanin pigments. This mechanism increases the absorption of the flavylium form and improves the red color intensity of the extracts. These results support our observations about the magnitude of the co‐pigmentation effects of each co‐pigment on anthocyanin extract.

Aromatic groups that are greater with freer –OH groups stabilize anthocyanins more than the others (Figueiredo et al., 1996). Hydrogen bonding and van der Waals forces between anthocyanins and organic acids are the two main stimulant factors for the co‐pigmentation process in this research, which Tan provides an appropriate opportunity for numerous formations of van der Waals interactions (Sun et al., 2010; Zhang et al., 2015). Sari et al. (2012) reported that the greatest bathochromic and hyperchromic shifts resulted at concentrations of 4, 3.5, 3, 2.5, 2, 1.5, 1, and 0.5 mg mL^−1^ of the rosemary polyphenolic extract and caffeic, sinapic, and ferulic acids, respectively. According to previous findings in the literature (Babaloo & Jamei, 2018; Malaj et al., 2013; Molaeafard et al., 2021; Zhang et al., 2015), the occurrence of hyperchromic effects and increase in the *A*_vis‐max_ follow an increase in the visible maximum wavelength due to the bathochromic shifts in the presence of co‐pigments, which leads to appearing brighter, bluer, more stable, and more durable colors than natural anthocyanins, which are in agreement with our results.

### 3.2 Co‐pigmentation as a function of increasing temperature

In this part of the experiments, the co‐pigments at their most effective concentration, 960 mg L^−1^, were compared with each other at increasing temperatures at pH 3.5. Figure 1j,k. indicates the visible absorption spectra of natural anthocyanin and anthocyanin complexes with various co‐pigments in the visible range of 400–700 nm. According to visible absorption spectra, the *A*_vis‐max_ of anthocyanin samples was repeatedly reduced in the presence of different co‐pigments due to the increase in temperature from 20°C to 100°C, which indicates the hypochromic effect in high‐temperature conditions. Figure 1l compares the spectra of anthocyanin with various co‐pigmented complexes at 100°C. In this figure, Tan had the least hypochromic effect, followed by Gal, Hbe, Caf, Mal, and, Acy, respectively. According to Dimitrić‐Marković et al. (2000), the weak interactions of anthocyanin‐co‐pigment complexes are eliminating due to the increase in the temperature levels of the reaction that happens toward the reactants. Moreover, it is believed that a sandwich‐shaped configuration caused by the π–π stacking in the co‐pigmentation complexes originated from anthocyanin molecules hinders a potential nucleophilic attack that may be caused by water to the flavylium cation chromophore (leading to colorless hydrated forms) physically (Trouillas et al., 2016). Accordingly, color stability is increased compared to natural anthocyanin.

**FIGURE 3:**
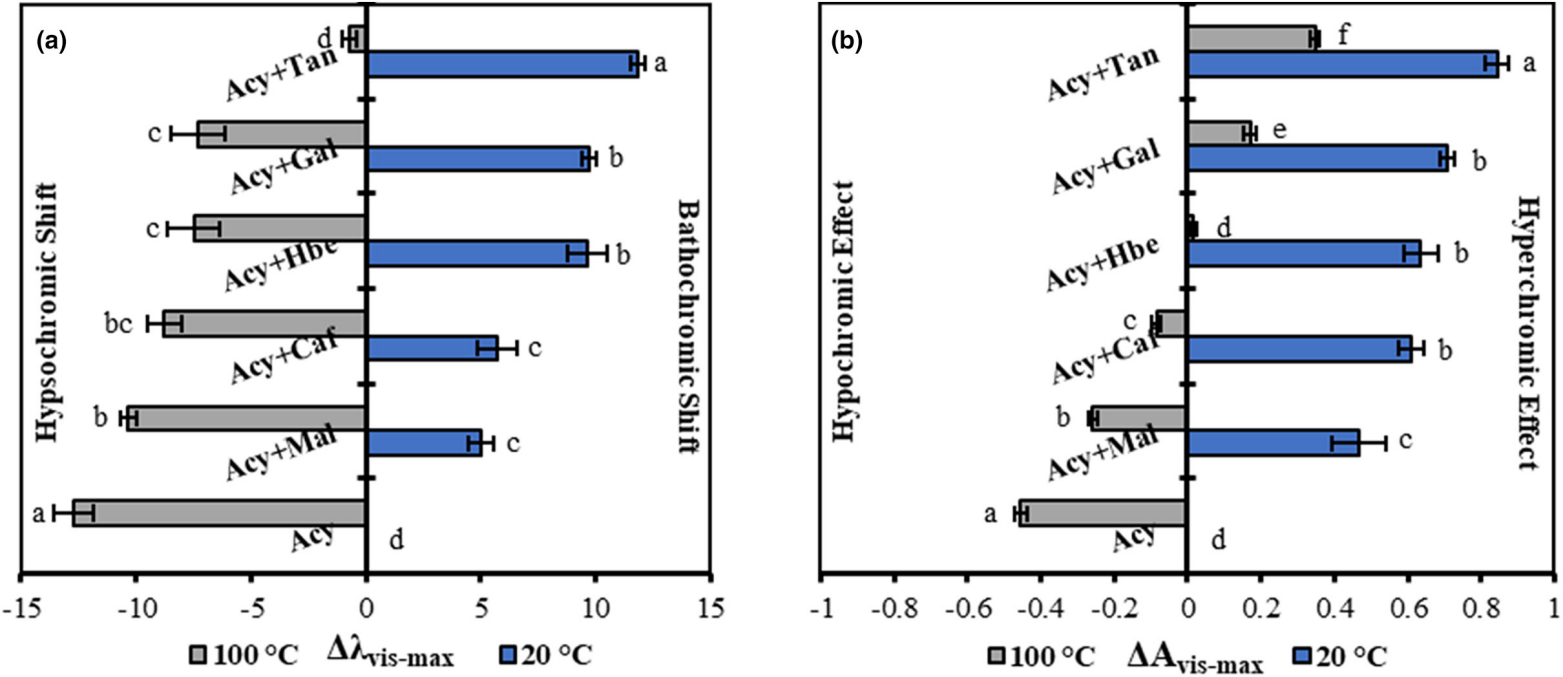
The magnitude of hypsochromic shift (a) and hypochromic effect (b) during interaction of co‐pigmentation and temperature effect on anthocyanins without and with co‐pigments (960 mg L^−1^) at 20°C and 100°C.

Also, the high temperature leads to a reduction in the visible maximum wavelength (*λ*_vis‐max_) and the occurrence of a hypsochromic shift in the visible absorption spectra. In Figure 3a,b, the correct values of the *A*_vis‐max_ and *λ*_vis‐max_ were compared at 20°C and 100°C for the accurate evaluation of hypsochromic shift and hypochromic effects between natural and various co‐pigmented anthocyanin samples. Figure 3a,b shows that the natural anthocyanin significantly (*p* ≤ .05) has the highest hypochromic effect (−0.455 ± 0.0164^a^ unit) and hypsochromic shift (−12.7 ± 0.864^a^ nm) compared to the anthocyanins‐co‐pigment complexes at 100°C. The lowest value of hypsochromic shift significantly (*p* ≤ .05) was related to Tan (−0.7 ± 0.327^d^ nm), followed by Gal (−7.33 ± 1.178^c^ nm), Hbe (−7.5 ± 1.132^c^ nm), Caf (−8.77 ± 0.728^bc^ nm), and Mal (−10.33 ± 0.327^b^ nm), respectively (Figure 3a). The positive values of Δ*A*_vis‐max_ in the Acy + co‐pigment complexes with Tan (0.347 ± 0.0108^f^ unit), Gal (0.1707 ± 0.0168^e^ unit), and Hbe (0.01667 ± 0.0103^d^ unit) indicated the hyperchromic effect in their presence, despite a large decrease in their *A*_vis‐max_ at 100°C (Figure 3b). Furthermore, Caf and Mal also had less hypsochromic and hypochromic shifts than anthocyanin samples without co‐piments. According to these results, the poorest and strongest Acy + co‐pigment interactions were formed in the presence of Mal and Tan, respectively. Moreover, we conclude that thermal degradation is prevented by co‐pigmentation of BCP anthocyanins because of the great variance (*p* ≤ .05) in the magnitude of hypochromic and hypsochromic shifts among anthocyanins and all co‐pigmented complexes. Also, the stability of co‐pigmented complexes in this study had differences, which, according to Trouillas et al. (2016), could be due to various structures of anthocyanins and co‐pigments as well as different environmental conditions such as temperature, which ultimately led to different affinity or binding energies between anthocyanins and co‐pigments. Some intermolecular interactions may occur due to the existence of co‐pigment aromatic rings, hydroxyl, or carbonyl‐O groups, resulting in a stable and durable interaction with anthocyanins (Sun et al., 2010; Zhang et al., 2015). Therefore, the findings show that the chemical composition of different co‐pigments, i.e. the absence of the aromatic ring in Mal, and the presence of several aromatic rings and abundant hydroxyl groups attached to them in Tan, may be the reason for their different effects.

### 3.3 Furfural formation and browning during heating time

**FIGURE 4:**
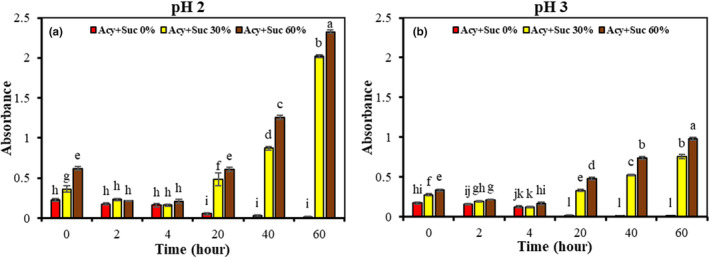
The effect of different concentrations of sucrose on the formation of polymeric color in pH 2 (a) and pH 3 (b) at 90°C in a period of 0–60 h in BCP anthocyanin samples.

In this study, the absorption of anthocyanins was measured at a wavelength of 520 nm because the maximum absorption of anthocyanins is at this wavelength. According to Figure 4a,b, the absorption decreased regularly in both pHs by increasing the heating time to 90°C in the samples containing 0% sucrose, which indicates the absence of furfural formation in the absence of sucrose. The decrease in absorption and red color were also observed in the samples containing 30% and 60% sucrose from 0 to 4 h. The increase in absorption in the mentioned samples compared to the samples without sucrose can be due to the expansion of the volume due to the addition of sucrose to anthocyanin samples. Also, the absorption of BCP anthocyanin samples containing sucrose increased during furfural and brown polymeric color formation at the range of 4–60 h; that was more at Suc 60% and pH 2 than Suc 30% and pH 3, which shows the heating solutions containing sucrose in strong acidity conditions cause severe hydrolysis of sucrose, which is also related to the concentration of sucrose. Türkyılmaz et al. (2019) reported that the degradation of anthocyanins due to the addition of sucrose can be followed by the formation of sucrose degradation products such as furfural due to the low pH of the samples (2.3) compared to higher pHs. They also concluded that the addition of sucrose causes an increase in the degradation rate constant and a decrease in the half‐life of both cyanidin 3‐O‐glucosylrutinozoide and cyanidin 3‐O‐rutinozoide isolated from sour cherries.

**FIGURE 5:**
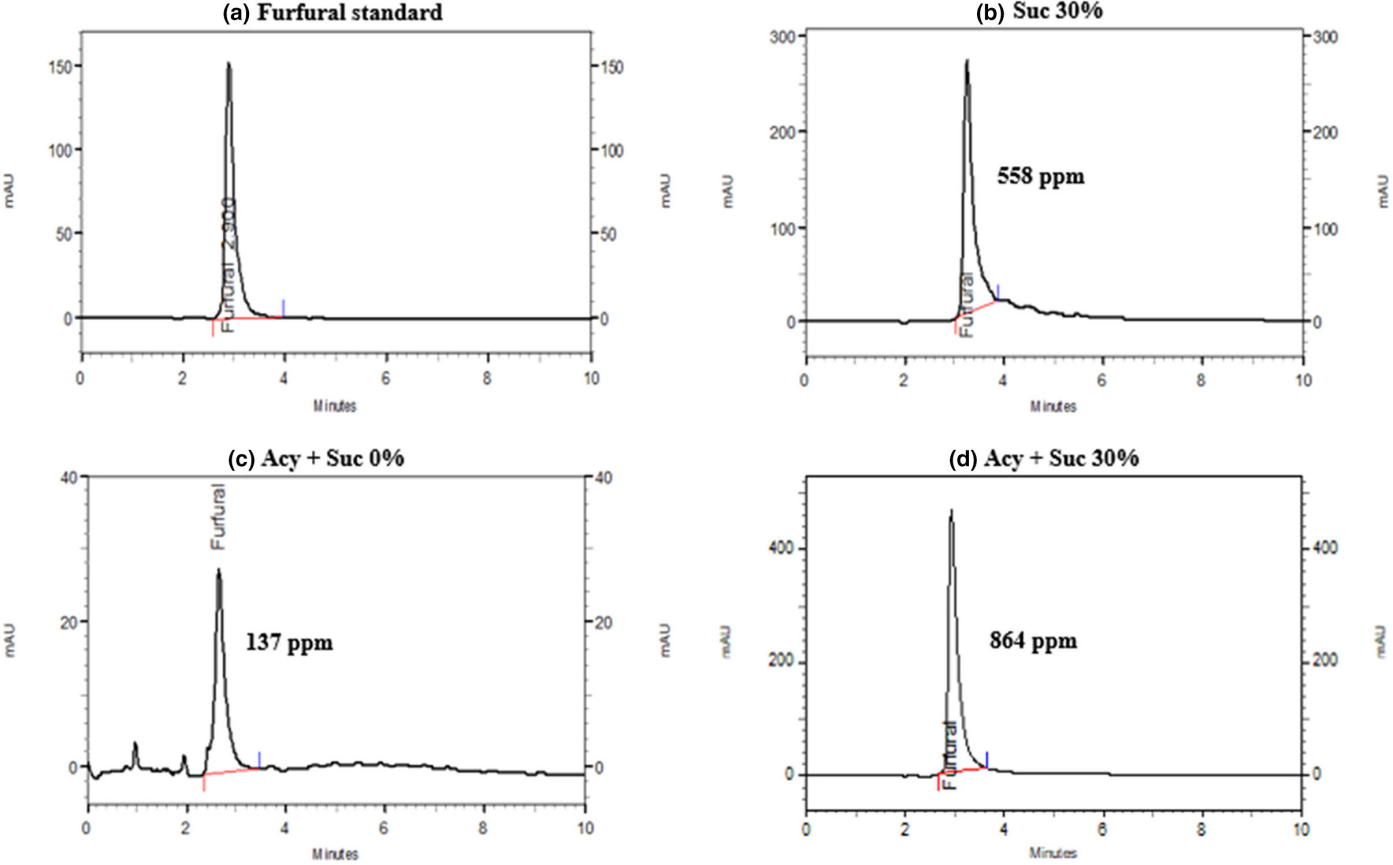
HPLC chromatogram of furfural content in furfural standard (a), sucrose 30% (b), Acy + sucrose 0% (c), and Acy + sucrose 30% (d) after 20 h of heating at 90°C.

Besides, the amount of formed furfural was measured in BCP anthocyanin samples (Figure 5c,d), and the resulting values were drawn in the form of a chromatogram, and the amount of formed furfural was expressed in ppm units. Also, the chromatograms related to the formation of furfural are shown in the furfural standard (Figure 5a) and Suc 30% samples (Figure 5b). The amount of furfural produced in the sample containing sucrose was higher than in the sample without sucrose. Also, the highest amount of furfural production was in the Acy + Suc 30% sample (864 ppm), followed by Suc 30% (558 ppm) and Acy + Suc 0% (137 ppm) samples. According to the suggestion of Tsai et al. (2005), the reaction between anthocyanin and furfural facilitates the decolorization of anthocyanin, which is covered by the products formed by heat. Prchalova et al. (2016) have observed that the amount of furfural and hydroxymethylfurfural formation increases during heating and increasing temperature in foods that contain sugar, such as apples and red fruits. Koulani et al. (2016) have also concluded that the furfural content in anthocyanin samples containing sucrose is higher than the samples without sucrose.

## AUTHOR CONTRIBUTIONS

**Shahrbanu Molaeafard:** Conceptualization (equal); data curation (lead); investigation (equal); methodology (equal); project administration (equal); supervision (supporting); visualization (equal); writing – original draft (lead); writing – review and editing (equal). **rashid jamei:** Conceptualization (equal); data curation (lead); investigation (lead); methodology (equal); project administration (equal); supervision (lead); visualization (equal); writing – original draft (supporting); writing – review and editing (equal). **Ahmad Poursattar Marjani:** Conceptualization (supporting); data curation (supporting); investigation (equal); methodology (supporting); project administration (supporting); supervision (supporting); visualization (equal); writing – original draft (supporting); writing – review and editing (equal).

## CONFLICT OF INTEREST STATEMENT

The authors declare that they have no conflict of interest.

## Data Availability

The findings supporting the present study, upon reasonable request, are available from the corresponding authors.
